# Comparison Between Distal Trans-radial Access and Conventional Trans-radial Access for Coronary Angiography

**DOI:** 10.7759/cureus.45081

**Published:** 2023-09-12

**Authors:** Manish Gupta, Vinit Kumar, Md Waziur Rahman, Swati Srivastava, Umeshwar Pandey, Santosh K Sinha

**Affiliations:** 1 Department of Cardiology, Laxmipat Singhania (LPS) Institute of Cardiology, Kanpur, IND; 2 Department of Cardiac Anesthesia, Laxmipat Singhania (LPS) Institute of Cardiology, Kanpur, IND

**Keywords:** anatomical snuffbox, puncture, radial artery occlusion, distal trans-radial access, conventional trans-radial access, coronary angiography

## Abstract

Background

Compared with a conventional wrist puncture for radial artery catheterization, a distal radial artery puncture has the advantage of reducing the incidence of radial artery occlusion (RAO).

Aim

The present study was designed to evaluate the feasibility of distal trans-radial access compared with conventional trans-radial access for coronary angiography.

Methods

A prospective, randomized, single-blinded, comparative study was conducted at a tertiary care center in India between December 2018 and November 2020. A total of 420 patients (aged >18 years) with signs and symptoms suggestive of coronary artery disease (CAD) and with a palpable radial artery in anatomical snuffbox were randomized into two groups. Group A comprised patients accessed at the distal trans-radial site, and Group B consisted of patients accessed at the conventional trans-radial site for coronary angiography. Baseline demographics, clinical history, and risk factors for CAD were documented. Procedural-related parameters and complications were compared between the two groups.

Results

The procedural success rate was non-significant between Group A and Group B (96% vs. 98%; p=0.38). Puncture in a single attempt was higher in Group B compared to Group A (92% vs. 78%; p<0.001). There was no significant difference between Group A and Group B for operation time (p=0.207), fluoroscopy time (p=0.183), and contrast volume (p=0.345). The rate of RAO was higher in Group B compared to Group A (13% vs. 2%; p<0.001). Radial artery hematoma/swelling at the puncture site between Group A (10%) and Group B (8%) was not significant (p=0.61). Post-procedural hemostasis time in Group A was 28 ± 7.86 minutes, and in Group B was 24 ± 6.23 minutes. Both post-procedural persistence of pain (p<0.001) and hand clumsiness (p<0.001) were significantly higher in Group B compared to Group A.

Conclusion

For coronary angiography, the distal trans-radial access site is a reliable and secure alternate access site.

## Introduction

Conventional trans-radial access is the standard method for coronary interventions, diagnosis, and treatment [1]. Compared to femoral access, it has a reduced risk of significant bleeding and a lower incidence of adverse cardiovascular events and vascular complications [2]. However, due to the low release of nitric oxide, endothelial damage, and reduced blood flow caused by the insertion of the sheath and catheter, conventional trans-radial access is more prone to complications such as radial artery spasm (RAS) and radial artery occlusion (RAO). These complications can extend the procedure time and increase patient discomfort [1,3]. The distal trans-radial access in anatomical snuffbox, proposed by Kiemeneij in 2017, has emerged as a promising alternative access to further reduce the risk for RAO [4,5]. The distal radial artery runs through the radial fossa (anatomical snuffbox) and anastomoses to complete the deep palmer arch with the ulnar artery. Proximal to this site, the radial artery has already given its branch to the superficial palmar arch. If any occlusion in the anatomical snuffbox occurs, tissue ischemia is prevented because of the maintenance of antegrade flow through the superficial palmar arch and the communicating collaterals [6,7]. Distal trans-radial access has several advantages over conventional radial access, including a lower risk of local complications, reduced hemostasis time, and enhanced patient and operator comfort [1,8,9]. However, there is a paucity of data examining the routine use of distal trans-radial access. To unravel the role of distal trans-radial access in current interventional practice, the present study was designed to evaluate the feasibility and complications of distal radial artery access compared to conventional radial access for coronary angiography.

## Materials and methods

Study design and population

This was a prospective, randomized, single-blinded, comparative study conducted at a tertiary care center in India between December 2018 and November 2020. The study was approved by the Institutional Ethics Committee of LPS Institute of Cardiology (Ec/bmhr/2018/21) on May 21, 2018, and written informed consent was obtained from all the patients before enrollment. A total of 420 patients were enrolled and randomly assigned into two groups: Group A comprised 210 patients in whom distal trans-radial access was used at anatomical snuffbox for coronary angiography, and Group B included 210 patients in whom conventional trans-radial access was used for coronary angiography. Inclusion criteria were (i) Patients aged >18 years; (ii) Patients with signs and symptoms suggestive of coronary artery disease (CAD); (iii) Patients willing to undergo coronary angiography; and (iv) Patients having palpable radial artery in anatomical snuffbox. Exclusion criteria were (i) Patients who had contraindications to contrast agents; (ii) Patients having forearm atrioventricular fistula for hemodialysis; (iii) Patients with a history of coronary artery bypass grafting in whom the radial artery was used as a graft; and (iv) Patients who had type III and type IV radial artery.

Data collection and methodology

Baseline demographics, clinical history, and risk factors for CAD were recorded for all the patients. Anthropometric measurements, chest X-ray, electrocardiogram, and 2D echocardiogram were recorded for each patient. Blood and serum investigations were carried out, including complete blood count, blood urea, serum creatinine, coagulation profile, and serum electrolytes. Coronary angiographic procedure-related parameters, including success rate, puncture in a single attempt, puncture time, operation time, fluoroscopy time, and contrast volume, were compared between distal trans-radial access (Group A) and conventional trans-radial access (Group B). The success rate was defined as the number of patients having successful visualization of coronary anatomy. Puncture time was defined as the time from holding a 21-gauge puncture needle in hand to successful radial artery cannulation, confirmed by arterial blood backflow from the radial sheath sidearm. Operation time was defined as time from prepping of skin to compression dressing. Incidences of procedure-related complications such as RAO, RAS, radial artery hematoma/swelling at the puncture site, post-procedural hemostasis time, the persistence of pain, and post-procedural hand clumsiness were recorded and compared between both groups.

Distal trans-radial artery puncture technique

The patient was positioned with the arm in a neutral position. A normal saline plastic bottle wrapped with a sterile cloth was put under the wrist's ventral aspect, and the patient was asked to hold a medium-sized ball of rolled gauge pieces. This served to keep the distal area open for access by separating the thumb and first finger, which ultimately increased patient comfort. The puncture of the distal trans-radial artery at the distal aspect of the palm was done with the snuffbox region facing upwards rather than the palmar aspect of the wrist. Once the routine disinfection of the skin was done, access was obtained. The distal radial artery was graded into four types based on findings of palpation as follows:

Type I 

Palpable artery without any effort with forceful thrust on the tip of the palmer aspect of the index finger, which is not suppressible with minimal force.

Type II 

Palpable artery without any effort, with forceful thrust on the tip of the palmer aspect of the index finger, which is suppressible with minimal force.

Type III

Palpable artery with effort with weak thrust on the palmer aspect of the index finger.

Type IV

Not palpable.

**Figure 1:**
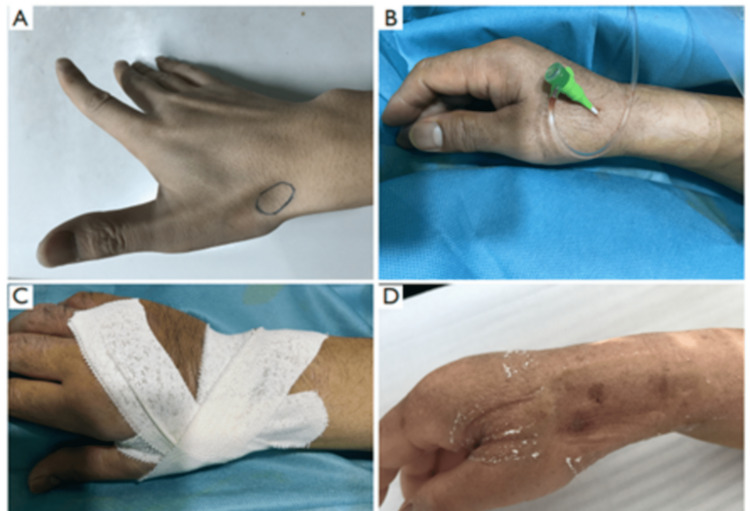
Distal trans-radial artery puncture technique. (A) Anatomical location of the puncture site; (B) Implantation of the 6F arterial sheath; (C) Application of the compression bandage with an elastic bandage post-intervention; (D) Recovery status of the puncture site.

Our study enrolled patients with type I and type II arteries. After subcutaneous injection of 1.5 mL xylocaine, the artery was punctured, preferably with a 21-gauge open needle, under an angle of 30°-45° from lateral to medial. The needle was directed to the point of the strongest pulse. Accessing the artery at the dorsum of the hand, distal to the tendon of the extensor pollicis longus muscle, was preferred where the artery is usually more superficial than at the snuffbox. A through-and-through puncture was avoided since the needle would touch the periosteum of the scaphoid or trapezium bones, which can be painful. The distal trans-radial artery puncture technique is represented in Figure 1.

Conventional trans-radial artery puncture technique

**Figure 2:**
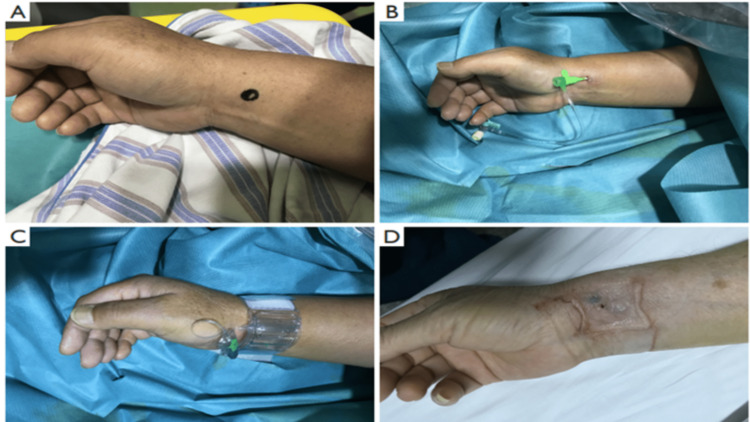
Conventional trans-radial artery puncture technique. (A) Recovery status of the puncture site; (B) Implantation of the 5F arterial sheath; (C) Application of the tourniquet compression bandage; (D) Recovery status of the puncture site post-intervention.

The right or left hand was set in the anatomical position, with the arm supinated and the wrist dorsiflexed. A roll gauze or towel was placed under the wrist for a comfortable extension. Subsequently, the access site was disinfected, and 1-2 ml of lignocaine was injected subcutaneously for local anesthesia. Then, the forearm radial artery was palpated to find the point of the strongest pulse. At an angle of 45°, the artery was punctured with a 21-gauge needle, and a 0.021" soft, flexible, metallic wire (Avanti trans-radial kit; Cordis Corp, USA) was then inserted through the needle. After pulling out the sheath, a compression device (Transparent Radial Artery Band; Terumo, Inc., Tokyo, Japan) was used for hemostasis. Patent hemostasis protocol was followed by placing a transparent radial artery band at the distal forearm with the placement of a small green box indicator present on the band proximal to the puncture site. Subsequently, 15-19 mL of air was used for the inflation of the band. The patency of the radial artery was checked by palpation and by the color of the palm every 20 minutes. The conventional trans-radial artery puncture technique is illustrated in Figure 2. Each procedure was performed by the same operator with experience in at least 1000 successful radial interventions and five years of experience in interventional cardiology for optimal comparison.

Statistical analysis

Data analysis was conducted using the SPSS software version 20 (IBM Corp., Armonk, NY, USA). Quantitative data were expressed as mean ± SD, while categorical data were represented as the number of patients (n) and percentage. The unpaired "t" test was used to compare the quantitative data of the two groups. For qualitative variables, the Chi-square test was employed. A p-value of <0.05 was considered statistically significant.

## Results

A total of 420 patients were enrolled in this study and randomized into two groups. Group A comprised 210 patients who underwent coronary angiography using distal trans-radial access at the anatomical snuffbox. Meanwhile, Group B consisted of 210 patients where conventional trans-radial access was utilized for coronary angiography. The mean age of patients in Group A was 55 ± 6.8 years, and in Group B was 54 ± 7.2 years (p=0.144). Males were predominant over females among Group A (60% vs. 40%; p=0.766) and Group B (58% vs. 41.9%; p=0.766). Risk factors, including hypertension (p=0.488), diabetes (p=0.517), hyperlipidemia (p=0.694), and smoking (p=0.769) were non-significant between Group A and Group B. Comparison of baseline demographics and risk factors of CAD among the study population are outlined in Table 1.

**Table 1:** Comparison of baseline demographics and risk factors of coronary artery disease among the study population.

Parameters	Group A (n=210)	Group B (n=210)	P-value
Age (years), (Mean ± SD)	55 ± 6.8	54 ± 7.2	0.144
Gender, (Male: Female)	126:84	122:88	0.766
Body mass index (kg/m²), (Mean ± SD)	23.47 ± 3.54	22.7 ± 4.71	0.059
Hypertension, n (%)	127 (60.6%)	119 (56.7%)	0.488
Diabetes, n (%)	66 (31.4%)	59 (28%)	0.517
Hyperlipidemia, n (%)	93 (44.2%)	88 (41.9%)	0.694
Smoking, n (%)	118 (56.1%)	114 (54.3%)	0.769

Procedural success rate was 96% in Group A and in Group B was 98% (p=0.38). The patients in Group A had a significantly lower number of radial artery punctures in the first attempt than those in Group B (78% vs. 92%; p <0.001). Puncture time in Group A was 1.6 ± 0.8 minutes, and in Group B was 1.2 ± 0.6 minutes (p=0.005). Operation time in Group A was 21.0 ± 2.9 minutes, and in Group B was 20.0 ± 3.3 minutes (p=0.207). Fluoroscopy time was higher in Group A than in Group B (4.2 ± 2.2 vs. 3.9 ± 2.4 minute; p=0.183). The mean contrast volume used was less in Group B (19 ± 5 mL) compared to Group A (21 ± 4 mL). A comparison of procedure-related parameters among the study population is demonstrated in Table 2.

**Table 2:** Comparison of procedure-related parameters among the study population.

Parameters	Group A (n=210)	Group B (n=210)	P-value
Procedural success rate, n (%)	202 (96%)	206 (98%)	0.38
Puncture in single attempt, n (%)	164 (78%)	193 (92%)	<0.001
Puncture time (min), n (%)	1.6 ± 0.8	1.2 ± 0.6	0.048
Operation time (min), Mean ± SD	21.0 ± 2.9	20.0 ± 3.3	0.207
Fluoroscopy time (min), Mean ± SD	4.2 ± 2.2	3.9 ± 2.4	0.183
Contrast volume (mL), Mean ± SD	21 ± 4	19 ± 5	0.345

Procedure-related complication such as RAO was observed in four (2%) patients in Group A and 27 (13%) patients in Group B (p <0.001). RAS was noted among three (1.5%) patients in Group A and among two (1%) patients in Group B (p=1.0). Post-procedural hemostasis time was found to be statistically significant between Group A and Group B (28 ± 7.86 minutes vs. 24 ± 6.23 minutes; p<0.001). Post-procedural persistence of pain (p<0.001) and post-procedural hand clumsiness (p<0.001) were found to be statistically significant among both groups. A comparison of procedure-related complications among the study population is represented in Table 3.

**Table 3:** Comparison of procedure-related complications among the study population.

Parameters	Group A (n=210)	Group B (n=210)	P-value
Radial artery occlusion, n (%)	4 (2%)	27 (13%)	<0.001
Radial artery spasm, n (%)	3 (1.5%)	2 (1%)	1.000
Radial artery hematoma/swelling at puncture site, n (%)	21 (10%)	17 (8%)	0.61
Post-procedure hemostasis time (min), Mean ± SD	28 ± 7.86	24 ± 6.23	<0.001
Post-procedure persistence of pain, n (%)	2 (1%)	29 (14%)	<0.001
Post-procedure hand clumsiness, n (%)	1 (0.5%)	19 (9%)	<0.001

## Discussion

In the past decade, distal trans-radial artery access at the anatomical snuffbox has emerged as a novel alternative to conventional trans-radial artery access for coronary angiography and interventions. In the present study, we compared the feasibility and safety of this novel distal trans-radial access with conventional trans-radial access for coronary angiography. In our study, the procedural success rate of distal trans-radial access (96%) was similar to that of conventional trans-radial access (98%). Echoing our findings, Sharma AK et al., in their study, also reported no superiority between distal and conventional radial access [10]. However, in the current study, we observed a significantly lower rate of successful punctures on the first attempt with distal trans-radial access (78%) compared to conventional trans-radial artery access (92%) (p=0.001). This aligns with the results of a previous study by Koutouzis et al., where the distal radial site was associated with an increased number of punctures (p<0.001) [11]. In our study, the puncture time was longer at the distal site (1.6 ± 0.8 minutes) compared to the traditional radial site (1.2 ± 0.6 minutes). This is consistent with a previous study by Koutouzis M et al., which reported a prolonged duration of cannulation at the distal site compared to the conventional radial site [11]. The increased rate of failure to cannulate the artery might be due to the increased tortuosity and angulations at the distal trans-radial site puncture. Conversely, the conventional trans-radial puncture site is usually on a straighter artery segment. In a meta-analysis by Feghaly et al., there was no statistically significant difference between distal radial access and conventional radial access in terms of fluoroscopy time and contrast doses [12]. Similarly, in our study, no significant differences were observed between the distal radial site and the conventional radial site regarding fluoroscopy time (p=0.183) and contrast volume (p=0.345).

Many studies have shown that distal trans-radial access results in a decreased rate of RAO [8,10-13]. This may be because the distal trans-radial access carries a lower risk of retrograde thrombus formation, given the collateral blood flow from the superficial palmar arch compared to the forearm trans-radial artery. Additionally, branches arising from the radial artery before the anatomic snuffbox form anastomoses with other wrist vessels, ensuring distal blood flow even in the presence of puncture-related ischemic complications [12]. In this study, a lower incidence of RAO was observed in the distal trans-radial access compared to the conventional trans-radial access (2% vs. 13%; p<0.001). However, the RAS rates were comparable between the distal trans-radial and conventional trans-radial sites (1.5% vs. 1%; p=1.0). Consistent with our findings, a previous study by Liang C et al. reported a lower incidence of RAO in distal trans-radial access (1.7% vs. 4.6%; p<0.001), though the RAS rates (3.4% vs. 2.6%, p= 0.354) were not significantly different between the two sites [1]. Liontou C et al. highlighted some disadvantages of distal trans-radial access. The smaller diameter of the distal trans-radial artery at the level of the anatomical snuffbox can increase the risk of vasospasm. The artery's tortuosity can also make it difficult or even impossible to advance the wire compared to the traditional wrist approach. In fact, distal trans-radial access is technically more demanding and often requires a steeper learning curve than conventional radial artery cannulation, even for those experienced in radial procedures [14].

In our study, higher post-procedural hemostasis time was noted in the distal trans-radial access compared to conventional trans-radial access (28 ± 7.86 minutes vs. 24 ± 6.23 minutes; p<0.001), which could be due to the less established occlusive device and unfamiliarity with the technique. On the contrary, in an earlier study by Vefalı V and Sarıçam E, hemostasis time was higher in traditional trans-radial access compared to the distal trans-radial access (20.23 ± 4.1 minutes vs. 11.85 ± 1.91 minutes; p<0.001) [15]. Post-procedural persistence of pain (p<0.001) and hand clumsiness (p<0.001) were both lower in the distal trans-radial access group compared to the conventional trans-radial access group. Our findings were comparable to a similar study by Sharma AK et al., who demonstrated the superiority of distal trans-radial access over the conventional trans-radial approach in terms of post-procedural persistence of pain (p<0.001) and post-procedural hand clumsiness (p<0.001) [10].

Study limitations

This study has some limitations. First, this was a single-center study. Second, the sample size was small. Third, puncture of the distal trans-radial artery can be challenging as its diameter is smaller than the radial artery. A steeper learning curve is required for its access, particularly when the anatomical snuffbox's pulse is weak.

## Conclusions

Conventional trans-radial access and distal trans-radial access techniques had comparable procedural success rates, RAS, and radial artery hematoma/swelling at the puncture site. Lower rates of complications such as RAO, post-procedural persistence of pain, and post-procedural hand clumsiness were observed among patients who underwent coronary angiography via distal trans-radial access. However, a puncture in a single attempt was prominent among patients accessed via a conventional trans-radial artery. Thus, distal trans-radial access is a feasible and safe alternative for coronary angiography and interventions. However, multicentric studies with larger sample sizes, including heterogeneous populations, are warranted to validate and generalize the findings of our study.
